# Late-long-term potentiation magnitude, but not Aβ levels and amyloid pathology, is associated with behavioral performance in a rat knock-in model of Alzheimer disease

**DOI:** 10.3389/fnagi.2022.1040576

**Published:** 2022-11-11

**Authors:** Metin Yesiltepe, Tao Yin, Marc D. Tambini, Lionel Breuillaud, Simone P. Zehntner, Luciano D’Adamio

**Affiliations:** ^1^Department of Pharmacology, Physiology & Neuroscience, New Jersey Medical School, Brain Health Institute, Jacqueline Krieger Klein Center in Alzheimer’s Disease and Neurodegeneration Research, Rutgers, The State University of New Jersey, Newark, NJ, United States; ^2^Biospective Inc., Montreal, QC, Canada

**Keywords:** amyloid precursor protein (APP), long term potentiation (LTP), β-secretase, amyloid-β, cognition, Alzheimer’s disease, knock-in rat, synaptic plasticity

## Abstract

Cleavage of Amyloid precursor protein by β- and γ-secretases lead to Aβ formation. The widely accepted pathogenic model states that these mutations cause AD via an increase in Aβ formation and accumulation of Aβ in Amyloid plaques. APP mutations cause early onset familial forms of Alzheimer’s disease (FAD) in humans. We generated *App*−Swedish (*App^s^*) knock−in rats, which carry a pathogenic *APP* mutation in the endogenous rat *App* gene. This mutation increases β-secretase processing of APP leading to both augmented Aβ production and facilitation of glutamate release in *App^s/s^* rats, via a β-secretase and APP−dependent glutamate release mechanism. Here, we studied 11 to 14-month-old male and female *App^s/s^* rats. To determine whether the Swedish *App* mutation leads to behavioral deficits, *App^s/s^* knock-in rats were subjected to behavioral analysis using the IntelliCage platform, an automated behavioral testing system. This system allows behavioral assessment in socially housed animals reflecting a more natural, less stress-inducing environment and eliminates experimenter error and bias while increasing precision of measurements. Surprisingly, a spatial discrimination and flexibility task that can reveal deficits in higher order brain function showed that *App^s/s^* females, but not *App^s/s^* male rats, performed significantly worse than same sex controls. Moreover, female control rats performed significantly better than control and *App^s/s^* male rats. The Swedish mutation causes a significant increase in Aβ production in 14-month-old animals of both sexes. Yet, male and female *App^s/s^* rats showed no evidence of AD−related amyloid pathology. Finally, *App^s/s^* rats did not show signs of significant neuroinflammation. Given that the APP Swedish mutation causes alterations in glutamate release, we analyzed Long-term potentiation (LTP), a long-lasting form of synaptic plasticity that is a cellular basis for learning and memory. Strikingly, LTP was significantly increased in *App^s/s^* control females compared to both *App^s/s^* sexes and control males. In conclusion, this study shows that behavioral performances are sex and *App*-genotype dependent. In addition, they are associated with LTP values and not Aβ or AD-related pathology. These data, and the failures of anti-Aβ therapies in humans, suggest that alternative pathways, such as those leading to LTP dysfunction, should be targeted for disease-modifying AD therapy.

## Introduction

Alzheimer’s disease (AD) is a common, age-related neurodegenerative disease characterized by the appearance of amyloid plaques and tau tangles. A small fraction of AD cases has early onset (<65 yo) and are due to inherited dominant mutations (FAD/EOAD); the vast majority of cases are sporadic and have a later onset (>65 yo, SAD/LOAD). Familial cases are caused by autosomal dominant mutations in either the *Aβ-Precursor Protein* (*APP*), *Presenilin-1* (*PSEN1*) or *Presenilin-2* (*PSEN2*) genes. LOAD cases do not have a monogenic component; however, genetic variants in some genes, such as *TREM2*, are associated with increased risk of LOAD (Guerreiro et al., 2013). Recently our laboratory has generated rat knock-in models of FAD/EOAD (Tambini et al., 2019; Tambini and D’Adamio, 2020a), LOAD (Ren et al., 2020, 2021; Tambini and D’Adamio, 2020b), as well as AD-related dementia (Yin et al., 2021). In these knock-in rats, AD-linked mutations are introduced into the rat genome. Thus, mutated genes are expressed under the control of endogenous regulatory elements at physiological levels, respecting the cell-type specific, temporal, and spatial expression patterns of the AD-linked gene.

APP is the protein from which Aβ, the major component of AD’s amyloid plaques, is derived after the sequential β-processing/γ-processing of APP (i.e., the amyloidogenic processing pathway). A rat knock-in model carrying the FAD/EOAD Swedish mutation of *APP* (*App^s^* rats) recapitulated the shift toward amyloidogenic processing of APP seen in AD patients with the same mutation, which was associated with increased release probability of synaptic vesicles in glutamatergic hippocampal neurons (Tambini et al., 2019). This finding of dysregulated glutamatergic signaling caused by the pathogenic mutations in the β-processing region of APP was consistent with the role of the juxta membranous domains of APP in binding synaptic vesicle machinery (Del Prete et al., 2014; Fanutza et al., 2015; Yao et al., 2019). Notably, these synaptic changes occur in peri-adolescent animals in the absence of amyloid plaque pathology, despite an increase in Aβ and APP-metabolites produced by β-cleavage (Tambini et al., 2019). Synaptic effects of the Swedish mutation were also described in a recent paper where the authors found that the APP Swedish mutation increases synapse numbers and synaptic transmission in isogenic human neurons cultures (Zhou et al., 2022). While synaptic dysfunction is a feature of AD, the defining clinical presentation of AD is progressive memory loss and cognitive decline. It is presently unclear whether the synaptic dysfunction seen in the *App^s^* rat model in the absence of amyloid plaque pathology will eventually lead to cognitive deficits similar to the clinical changes in behavior that characterize AD.

To uncover associations among diverse functional changes prompted by the Swedish mutation, we analyzed behavior, APP processing, neuroinflammation, brain pathology and synaptic plasticity in 11–14 month-old *App^s/s^* rats and *App^h/h^* rats. *App^h/h^* rats, which serve as our control group, carry the humanized Aβ sequence. This control knock-in rat line was generated because: (1) aggregated or oligomeric forms of Aβ are by and large considered the main pathogenic entity in AD; (2) human Aβ differs from rat Aβ by 3 amino-acids and has higher propensity to form toxic Aβ species as compared to rodent Aβ. In *App^s/s^* rats, together with the Swedish mutations, we also introduced mutations to humanize the rat Aβ sequence. Thus, both control *App^h/h^* and mutant *App^s/s^* rats studied here produce human Aβ, which replaces rat Aβ.

Behavioral analysis was performed using the IntelliCage platform (Endo et al., 2011; Wu et al., 2017), an automated behavioral testing system, which was chosen for several reasons: (1) Its large, centralized cage design allows for high throughput testing. (2) Behavioral assessment in socially housed animals reflects a more natural, less stress-inducing environment. (3) Automation eliminates experimenter error and bias while increasing precision of measurements. (4) The standardized housing and testing equipment increases inter-laboratory reproducibility. A similar system for mice has been used for studying the behavior of many models of neurodegeneration, including a knock-in mouse model of Swedish *App* alone and in combination with other *App* mutations (Masuda et al., 2016). To establish a baseline in performance, we have recently used the platform to test the learning and memory of control *App^h/h^* rats (Pham et al., 2022). Following the behavioral assessment, biochemical analysis of changes in APP-metabolism and neuroinflammation, and histopathological analysis of plaque and tangle status, gliosis, and neurodegenerative changes was performed. Finally, Long-Term Potentiation (LTP), a long-lasting form of synaptic plasticity described at glutamatergic synapses throughout the brain and one of the most attractive cellular models for learning and memory, was analyzed in a different cohort of 12-month-old animals.

## Materials and methods

### Experimental animals

All experiments were done according to policies on the care and use of laboratory animals of the Ethical Guidelines for Treatment of Laboratory Animals of the NIH. Relevant protocols were approved by the Rutgers Institutional Animal Care and Use Committee (IACUC) (Protocol #201702513). All efforts were made to minimize animal suffering and reduce the number of rats used.

### Rat genotyping

The insertion in *App* exon 16 of humanizing mutations (*App^h^* allele) and the humanizing + Swedish mutations (*App^s^* allele) were verified by DNA sequencing of genomic DNA PCR products that include exon 16 as previously reported. Briefly, tail tissue was digested in 300 μl lysis buffer (100 mM Tris, 5 mM EDTA, 0.2% SDS, 200 mM NaCl, pH 8.0) plus 3 μl of 20 μg/ml protease K at 55°C overnight. One hundred μl of a 7.5M Ammonium Acetate solution was added to each sample to precipitate protein, samples were mixed by vortexing for 30 s and centrifuged at 15,000 × *g* for 5 min. Supernatant was mixed with 300 μl Isopropanol and centrifuged at 15,000 × *g* for 5 min. to precipitate genomic DNA. The DNAs pellet was desalted with 70% ETOH and was dissolved in water for PCR and sequencing. The rats studied here were obtained by crossing *App^s/h^* male and females. Ten breeding pairs were used. To avoid litter effects, for each cohort no more than 2 females and 2 male rats from each breeding pair were used.

### Behavioral experiments and analysis

Learning and memory were analyzed in 11–13-month-old rat. Prior to behavioral analyses, male rats were housed 2 per cage and female rats were housed 4 per cage under controlled laboratory conditions with a 12-h dark/light cycle (dark from 7 pm to 7 am), at a temperature of 25 ± 1°C. One month before testing, rats were lightly anesthetized with isoflurane, tagged subcutaneously with radio frequency identification transponders (RFID). Rats had free access to standard rodent diet and tap water while in traditional housing and were monitored for dehydration during periods of water restriction during behavioral analysis. The IntelliCage for Rats (NewBehavior AG) was used to collect behavioral data. The IntelliCage system is a fully automated approach to cognitive assessment in a social context with user-designed programs (TSE Systems, Bad Homburg, Germany). Each IntelliCage consists of a large common space with bedding, 4 black shelters in the center, and *ad libitum* food windows on two sides. At each corner of the IntelliCage is an operant chamber connected to the central common space by an opening that can detect tagged rats by RFID. The openings lead to remote-controlled doors which can detect nosepokes and slide open to allow access to drinking bottles. All visits are recorded remotely by IntelliCage. Rats are housed in the IntelliCage for the total duration of the experiment, with 8 rats per IntelliCage and 4 IntelliCages running simultaneously. IntelliCages are kept in a 12-h dark/light cycle (7 am–7 pm) and at a temperature of 25 ± 1°C. Each experiment lasts 18 days total, after which the IntelliCage is cleaned before a new cohort is introduced. Briefly, the program timeline consisted of the following parts. A period during which the animals may freely explore the IntelliCage and acclimate to a daily period of restricted water access during a time window (8:00–11:00 pm) called the *drinking session*. A period consisting of Place Learning program, during which every animal is assigned a drinking corner during a drinking session. A period consisting of Place Learning with Corner Switch program, during which, each rat is assigned an initial drinking corner, as in Place Learning. Every 45 min, the drinking corner switches to another adjacent corner. A period consisting of more complex sequencing programs involving a rule that governs the designation of drinking corners based on animal activity during a drinking session. Below, is a more detailed description of the IntelliCage programs used in these experiments.

Free adaptation (5 day): The rats may drink water ad libitum and explore the IntelliCage, familiarizing themselves with its layout; all bottle access doors open in response to any corner visit.

Nosepoke adaptation (2 days): The rats learn they must activate a nosepoke sensor to open a water access door at any corner for 7 s; this nosepoke mechanic remains active for every program hereafter.

Time adaptation (3 days): The rats may only drink during the drinking session (between 8 pm and 11 pm) at any corner.

Place learning (2 days): The rats may only drink during the drinking session at a corner assigned to each of them; these assigned corners are considered correct, and the non-assigned corners are considered incorrect. Only two rats share the same correct corner at any time.

Place learning with corner switch (2 days): Each rat is assigned an initial correct corner where it can drink during the drinking session, as in place learning, with the other corners being incorrect. Every 45 min, the correct corner designations are switched according to the cycle (1- > 3- > 4- > 2[- > 1]). If corner 2 were the initial correct corner, the cycle would be shifted over once (2- > 1- > 3- > 4[- > 2]). After the first switch, the positions of the incorrect corners adjust accordingly. By the first 45-min block of the next drinking session, the correct corner will have returned to its initial location. A *phase* refers to a 45-min block during the drinking session in this program. The end of a phase marks when a corner switch occurs. Ultimately, all 4 corners will be consecutively, but not concurrently, “correct” for a given rat during a 3-h drinking window. Only two rats share the same correct corner at any time.

Behavioral sequencing (3 days): Rats are again assigned to a correct corner, and the rats must alternate between drinking at this initial learned correct corner and the opposite corner during the drinking session. Thus, the conditions of the corners in the assigned diagonals alternate between correct and previously correct. The switch is triggered by one nosepoke in the correct corner, which becomes previously correct, while the corner diagonal now becomes correct. Visits to corners in the non-assigned diagonal are considered lateral visits. Only two rats share the same correct corner at any time. Table 1 shows a summary of the timeline.

**TABLE 1:** IntelliCage programs timeline overview.

Number of days	Task
5	Free adaptation
2	Nosepoke adaptation
3	Time adaptation
2	Place learning
2	Place learning with corner switch
3	Behavioral sequencing

*Learning curves, area under the learning curve (AUC), inclusion/exclusion criteria and statistical analysis of AUC*. To visualize learning of all the animals of same sex and genotype, we charted the fractional accumulation of correct visits over the course of each drinking session. To assess task performance quantitatively, we used the area under the learning curves of individual animals. With the resulting curves and AUCs, we can qualitatively compare task performance according to drinking session, sex, and genotype. These methods have been previously described in detail (Pham et al., 2022). Data were tabulated and graphed using Microsoft Excel and GraphPad Prism.

For analysis of Place Learning data, a visit to the assigned corner was counted as “correct” and all other visits were recorded to obtain the total number of visits. For each animal the fractional accumulation of “correct” visit, i.e., all “correct” visits expressed as a fraction of total number of all visits, was plotted against time to produce a learning curve. This analysis was performed separately for each day of the 2 days of the Place Learning experiment.

For analysis of Place Learning with Corner Switch data, a similar analysis was performed. Each visit was scored “correct” only if the “correct” assigned corner was visited. The identity of each “correct” corner would then change every 45 m as described above. For each animal, the fractional accumulation of “correct” visit, i.e., all “correct” visits expressed as a fraction of total number of all visits, was plotted against time to produce a learning curve. This analysis was performed separately for each day of the 2 days of the Place Learning with Corner Switch experiment.

For analysis of Behavioral Sequencing data, each visit was scored “correct” only if the “correct” assigned corner was visited. The identity of the “correct” corner would then change after every visit-limited nosepoke as described above. For each animal, the fractional accumulation of each visit was plotted against time to produce a learning curve. This analysis was performed separately for each day of the 3 days of the Behavioral Sequencing experiment.

During drinking sessions, animals may visit corners infrequently or not at all. Animals that did not make more than 25 visits during a drinking session in a learning and memory task were excluded from the analysis of that session. Animals that did not make sufficient visits for two consecutive drinking sessions were removed from the IntelliCage the following morning and allowed to drink water freely for an hour before being returned to the IntelliCage. Animals that died at any point during the timeline were excluded from the analyses of the current drinking session and, for obvious reasons, from all subsequent drinking sessions. For all behavioral experiments, a total of 18 female *App^h/h^*, 19 male *App^h/h^*, 10 female *App^s/s^*, and 11 male *App^s/s^*, rats were used. No animals met the exclusion criteria of <25 visits per drinking session, and none died during the behavioral experiments.

To quantitatively measure differences in task performance, individual learning curves generated for each animal were analyzed using Microsoft Excel and GraphPad Prism software and expressed as mean ± SEM. Differences were assessed by Two-way ANOVA. Data showing statistical significance by Two-way ANOVA were subsequently analyzed by Šídák’s multiple comparisons tests. *p* < 0.05 was considered statistically significant.

### Rats brain proteins preparation, ELISAs, dot blots, and western blots

These procedures were performed as previously described (Tamayev et al., 2010; Tambini and D’Adamio, 2020a,b; Yin et al., 2021). Briefly, rats were anesthetized with isoflurane and perfused via intracardiac catheterization with ice-cold PBS. Brains were extracted and homogenized with a glass-teflon homogenizer in 250 mM Sucrose, 20 mM Tris-base pH 7.4, 1 mM EDTA, 1 mM EGTA plus protease and phosphatase inhibitors (ThermoScientific). All steps were carried out on ice. Homogenates were solubilized with 1% NP-40 for 30 min rotating and spun at 20,000 *g* for 10 min. Supernatants were collected and protein content was quantified by Bradford. To prepare s100 and p100 fractions for dot blot analysis, homogenates were centrifuged at 100,000 *g* for 1 h to collect supernatants (s100 fraction). The left pellet of the 100,000 *g* centrifugation step was solubilized with 1% NP-40 with 30 min rotating and centrifuged at 20,000 *g* for 10 min to collect solubilized p100.

For *western blots (WB) analyses*, proteins were diluted with PBS and LDS Sample buffer-10% β-mercaptoethanol (Invitrogen NP0007) and 4.5M urea to 1 μg/μl, loaded on a 4–12% Bis-Tris polyacrylamide gel (Biorad 3450125), and transferred onto nitrocellulose at 25 V for 7 min using the Trans-blot Turbo system (Biorad). Blotting efficiency was visualized by red Ponceau staining on membranes. Membranes were blocked 45 min in 5%-milk (Biorad 1706404), washed extensively in PBS/Tween20-0.05%, and the following primary antibodies were applied: Y188 (APP-C-terminus, Abcam ab32136, 1:1,000 dilution, O/N at 4°C), anti-GAPDH (Origene TA308884, 1:10,000, O/N at 4°C), DA9 (1:1,000, O/N at 4°C), PHF1 (1:1,000, O/N at 4°C), CP13 (1:1,000, O/N at 4°C). DA9, CP13, and PHF1 have been produced Dr. Peter Davies’ lab, AECOM and Feinstein Institute for Medical Research, and have been widely used, published and validated. Either anti-mouse HRP-conjugated (Southern Biotech 1031-05) or a 1:1 mix of anti-rabbit HRP-conjugated (Southern Biotech, OB405005) and anti-rabbit HRP-conjugated (Cell Signaling, 7074), were diluted 1:1,000 in 5% milk and used against mouse and rabbit primary antibodies for 45 min. at RT, with shaking. Blots were developed with West Dura ECL reagent (Thermo, PI34076) and visualized on a ChemiDoc MP Imaging System (Biorad).

For *dot-blot analysis* 5 μg of material was directly spotted with a p20 pipette on a nitrocellulose membrane. Dot membrane was also visualized by red Ponceau after it was totally dried. Membranes were blocked with 5% Non-fat dry milk (Bio-Rad, 1706404), washed with PBS/Tween-20 (0.05%) and applied A11 primary antibody (1:2,000, shared by Rakez Kayed’s Lab), which is diluted in blocking solution (Thermo Fisher Scientific, 37573) 1 h at RT. Membranes were washed 3 × 10 min and subsequently against anti-rabbit HRP-conjugated (1:1 mix of SouthernBiotech, OB405005 and Cell Signaling Technology, 7074) at 1:1,000 dilution for 45 min with shaking at RT. Membranes were developed with West Dura ECL reagent (Thermo Fisher Scientific, PI34076) and visualized on a ChemiDoc MP Imaging System (Bio-Rad). Signal intensity was quantified with Image Lab software (Bio−Rad). Data were analyzed using Prism software.

*ELISA.* For analysis of Aβ40, Aβ42, sAPPα and sAPPβSw, the following Meso Scale Discovery kits were used: Aβ40, and Aβ42 were measured with V-PLEX Plus Aβ Peptide Panel 1 6E10 (K15200G); sAPPα was measured with sAPPα (K15120E); sAPPβ-Sw was measured with sAPP Swedish sAPPβ (K151BUE). Measurements were performed according to the manufacturer’s recommendations. Plates were read on a MESO QuickPlex SQ 120. For analysis of Aβ43, IBL Human Amyloidβ (1–43) (FL) Assay Kit (27710) was used according to the manufacturer’s recommendations. Data were analyzed using Prism software.

### Immunohistochemistry

Rat brain tissue was fixed and stored in 70% ethanol after transcardiac perfusion with PBS and 4% paraformaldehyde fixative. All tissues were dehydrated through graded ethanol and xylene, infiltrated with paraffin wax, and embedded in paraffin blocks. Sections were cut on a rotary microtome at the thickness of 5 μm, floated on a water bath and mounted on glass slides. Slides were manually deparaffinized and rehydrated before the automated immunohistochemistry (IHC). Slides initially underwent antigen retrieval, by one of the following methods, heat-induced epitope-retrieval (HIER) or formic acid (FA) treatment. HIER was performed by incubation in a citrate buffer (pH 6.0) (Abcam, ab93678) and heating to 100°C for a period of 60 min. FA treatment was a 10-min incubation in 80% FA (Sigma, F0507), followed by washing in tris-buffered saline-Tween 20. All IHC studies were performed at room temperature on a Lab Vision Autostainer 360 (Thermo). Briefly, slides were incubated sequentially with hydrogen peroxide for 5 min, to quench endogenous peroxidase, followed by 5 min in a protein block (Abcam, ab156024), and then incubated with primary antibodies (see Table 2) in antibody diluent (Abcam, ab64211). Antibody binding was amplified using the appropriate secondary reagents (Jackson) (20 min), followed by a horseradish peroxidase conjugate (Jackson) (20 min), and visualized using the aminoethyl carbazole chromogen (Abcam, ab64252) (20 min). All IHC sections were counterstained with Acid Blue 129 (Sigma, 306496) and mounted with an aqueous mounting medium.

**TABLE 2:** Primary and amplification antibodies used for IHC.

Target	Antibody	Antigen retrieval	Dilution	Secondary & amplification
Neurons	NeuN, Mouse monoclonal A60, Millipore	Citrate HIER	1:3,000	RbαM & GtαRb-HRP
Amyloid β	1–16 and 17–24 Amyloid β Mouse monoclonal 6E10 and 4G8, Biolegend	80% Formic Acid	1:1,000 1:1,000	RbαM & GtαRb-HRP
Microglia	Iba1, Rabbit polyclonal, Wako	Citrate HIER	1:200	GtαRb-HRP
Astrocytes	GFAP, Rabbit polyclonal, Thermo Scientific	Citrate HIER	1:200	DkαRb-bio & SA-HRP
Phospho-tau	Phospho-tau, AT8, Mouse monoclonal, Thermo Scientific	Citrate HIER	1:1,000	HαM-bio & SA-HRP

∝, anti; bio, biotin; Dk, donkey; Gt, goat; HIER, heat induced antigen retrieval; H, horse; HRP, horseradish peroxidase; M, mouse; Rb, rabbit; SA, streptavidin.

### Hippocampal electrophysiology experiments

Rats were anesthetized with isoflurane (Covetrus, OH) and intracardiac perfusion was performed using ice-cold cutting solution containing 120 mM choline chloride, 26 mM NaHCO_3_, 15 mM D-Glucose, 2.6 mM KCl, 1.25 NaH_2_PO_4_, 1.3 mM ascorbic acid, 7 mM MgCl_2_, and 0.5 mM CaCl_2_. The brains were removed from the skull and rapidly placed in ice-cold cutting solution bubbled with 95% O_2_/5% CO_2_. Coronal brain slices (300 μm thickness) were prepared using Vibratome VT1200S (Leica, Germany). Hippocampal formations were dissected using a microsurgical knife (Electron Microscopy Sciences, CA) and hippocampal slices were incubated for 1 h in ACSF containing 124 mM NaCl, 26 mM NaHCO_3_, 10 mM D-Glucose, 3 mM KCl, 1 mM MgSO_4_, 1.25 mM KH_2_PO_4_ and 2 mM CaCl_2_, bubbling with 95% O_2_/5% CO_2_ at 30°C. Later hippocampal slices were transferred to the multielectrode dish (MED-515A, Alpha MED Scientific Inc, Japan) with a 150μm interelectrode distance. The chamber was perfused with oxygenated ACSF at a flow rate of 2 mL/min, at 32°C. Basal field excitatory postsynaptic potentials (fEPSP) were generated by stimulating Schaffer collaterals at 0.05 Hz and all recordings were done in stratum radiatum layer of CA1 hippocampal region. For input/output curves (I/O) the stimulation strength was increased from -5 to -80 μA in steps of 5 μA. The threshold stimulus was determined as the stimulus strength needed to generate 30–40% of maximum fEPSP amplitude during I/O curve recordings. In paired-pulse experiments, fEPSP responses were elicited by a double stimulus with interpulse intervals of 20, 40, 80, 200, and 500 ms. The percentage of facilitation was the slope of the second fEPSP response divided by the slope of the first fEPSP response. The long-term potentiation (LTP) was induced after 15 min of baseline recording with a ⊖-burst stimulation. ⊖-burst stimulation parameters; Burst = 4 pulses with threshold stimulus at 100 Hz (10 ms pulse-intervals). This burst was repeated 10 times at 5 Hz and named as a train (200 ms burst-intervals). 4 trains of 10-bursts were administered at 10 s intervals (in total 40 bursts were applied). LTP was analyzed in 3 different phases; short term potentiation (STP, 11–20 m), early-LTP (E-LTP, 51–60 m) and late-LTP (L-LTP, 111–120 m). Data were filtered at 1 kHz, digitized at 20 kHz and analyzed with Mobius software (Alpha MED Scientific Inc, Japan).

### Statistical analysis

Data were analyzed using GraphPad Prism software and expressed as mean ± SEM. Statistical tests used to evaluate significance and statistical data are shown in Figure legends. Significant differences were accepted at *P* < 0.05.

## Results

### Behavioral sequencing test of *App^h/h^* and *App^s/s^* rats reveals sex- and genotype-dependent higher order executive functions deficits

To characterize the effect of the *App*-Swedish mutation on learning and memory, the IntelliCage platform was used to analyze *App^h/h^* and *App^s/s^* rats. The IntelliCage is automated, allowing for accurate, unbiased recording and scoring, while simultaneously avoiding the potential influence of the human experimenter’s presence on animal behavior during testing. The rats are tested while housed with other seven rats, a social setting more reflective of their biological habitat and one that allows for a higher throughput of subjects in a uniform setting that also increases inter-laboratory reproducibility. Though relatively new, this experimental system has been used to interrogate behavior in many studies, including in a younger cohort of *App^h/h^* knock-in rats (Pham et al., 2022) and in an *App* knock-in mouse model similar to the *App^s/s^* rats used in this study (Masuda et al., 2016).

A total of 58 rats were tested: 18 female and 19 male *App^h/h^* rats, and 10 female and 11 male *App^s/s^* rats. All rats were subjected to a series of adaption programs designed to habituate the rats sequentially to the IntelliCage setting (Free adaptation, 5 days), to the use of a nosepoke to access water (Nosepoke adaptation, 2 days), and finally to the restriction of water access to a 3 h period (Time adaptation, 3 days). Post adaptation, rats were then subjected to a series of three tests, “Place Learning,” “Place Learning with Corner Switch,” and “Behavioral Sequencing” which carried forward the water-access restrictions learned during adaption. All rats tested were used for data analysis, and no rats died during the experiments.

**FIGURE 1:**
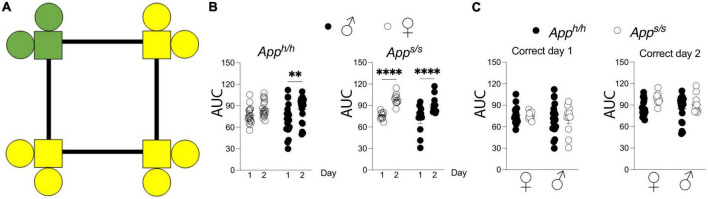
Place learning analysis of *App^h/h^* and *App^s/s^* rats. **(A)** Schematic of operant corner, with “correct” corner in green and incorrect in yellow. **(B)** Area under the “correct” learning curve analysis for individual rats stratified by each day of the drinking session. Data are represented as mean ± SEM and were analyzed by two–way RM ANOVA followed by *post-hoc* Sidak’s multiple comparisons test when ANOVA showed significant differences (*App^h/h^* rats: day factor, *F*_(1, 35)_ = 14.23, *P* = 0.0006; sex factor, *F*_(1, 35)_ = 0.5230, *P* = 0.4744; day × sex interaction, *F*_(1, 35)_ = 0.5370, *P* = 0.4686; *post-hoc* Sidak’s multiple comparisons test, females day 1 vs. day 2 *P* = 0.0806, males day 1 vs. day 2 *P* = 0.0054. *App^s/s^* rats: day factor, *F*_(1, 19)_ = 74.29, *P* < 0.0001; sex factor, *F*_(1, 19)_ = 1.784, *P* = 0.1974; day × sex interaction, *F*_(1, 19)_ = 0.3865, *P* = 0.5415; *post-hoc* Sidak’s multiple comparisons test, females day 1 vs. day 2 *P* < 0.0001, males day 1 vs. day 2 *P* < 0.0001). **(C)** Area under the “correct” learning curve analysis for individual rats stratified by sex and genotype. Data are represented as mean ± SEM and were analyzed by two–way ANOVA followed by *post-hoc* Sidak’s multiple comparisons test when ANOVA showed significant differences. Correct day 1: sex factor, *F*_(1, 54)_ = 1.358, *P* = 0.2490; genotype factor, *F*_(1, 54)_ = 0.0246, *P* = 0.8760; sex × genotype interaction, *F*_(1, 54)_ = 0.0012, *P* = 0.9719. Correct day 2: sex factor, *F*_(1, 54)_ = 1.515, *P* = 0.2238; genotype factor, *F*_(1, 54)_ = 1.998, *P* = 0.0960; sex × genotype interaction, *F*_(1, 54)_ = 0.9083, *P* = 0.3448). We tested 18 female *App^h/h^*, 19 male *App^h/h^*, 10 female *App^s/s^*, and 11 male *App^s/s^*. No animals met the exclusion criteria of <25 visits per drinking session, and none died during the behavioral experiments. ^**^P < 0.01, and ^****^P < 0.0001.

Spatial learning was assessed with the “Place learning” and “Place Learning with Corner Switch” programs. In “Place learning” each rat was assigned a “correct” corner which was held constant during the 3 h drinking windows across two days (Figure 1A). For each individual rat, a learning curve was generated by the plotting of accumulation of “correct” visits as a fraction of total visits over time. Areas under the learning curve were then derived and used to make comparisons between performances during the different days of the “Place Learning” trial, and between sex-dependent and genotype-dependent effects on task performance (Figures 1B,C). As expected, the rate of performance improved over time, with statistically significant improvements seen on the second day of the Place Learning task for all groups, with the exception of female *App^h/h^* rats whose performance improved but did not reach statistical significance (Figure 1B). Within each day, task performance was not significantly different between sexes and genotypes (Figure 1C).

**FIGURE 2:**
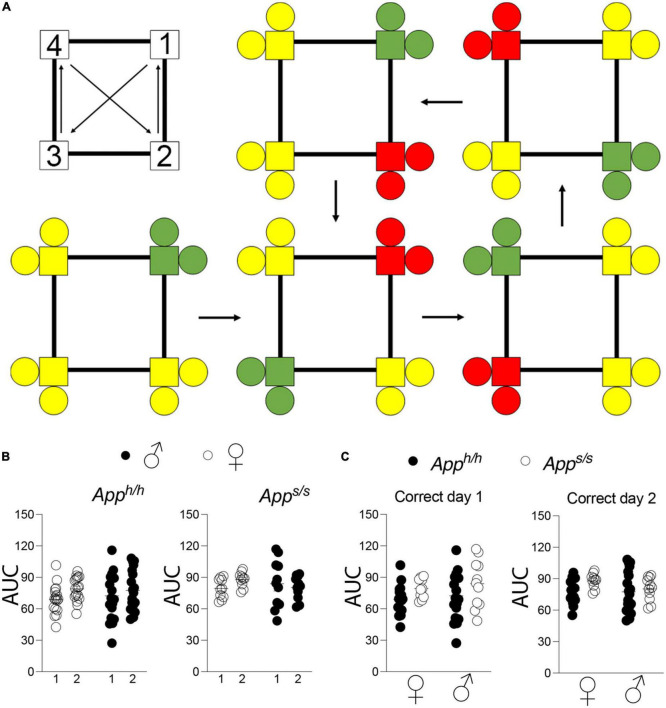
Place learning with corner switch analysis of *App^h/h^* and *App^s/s^* rats. **(A)** Schematic of corner switching program on top left, with a 45 min between each corner switch. For rats assigned to corner 1, bottom left, green indicates “correct” corner which then alternates to an adjacent corner every 45 min (arrows), with yellow indicating “incorrect” corners and red indicating “incorrect, previously correct” corner. **(B)** Area under the “correct” learning curve analysis for individual rats stratified by each day of the drinking session. Data are represented as mean ± SEM and were analyzed by two–way RM ANOVA followed by *post-hoc* Sidak’s multiple comparisons test when ANOVA showed significant differences (*App^h/h^* rats: day factor, *F*_(1, 35)_ = 3.540, *P* = 0.0683; sex factor, *F*_(1, 35)_ = 0.05113, *P* = 0.8224; day × sex interaction, *F*_(1, 35)_ = 1.041, *P* = 0.4525. *App^s/s^* rats: day factor *F*_(1, 19)_ = 0.3123, *P* = 0.5828; sex factor, *F*_(1, 19)_ = 0.1210, *P* = 0.5059; day × sex interaction, *F*_(1, 19)_ = 1.900, *P* = 0.1840). **(C)** Area under the “correct” learning curve analysis for individual rats stratified by sex and genotype. Data are represented as mean ± SEM and were analyzed by two–way ANOVA followed by *post-hoc* Sidak’s multiple comparisons test when ANOVA showed significant differences. Correct day 1: sex factor *F*_(1, 54)_ = 0.6217, *P* = 0.4339; genotype factor, *F*_(1, 54)_ = 2.078, *P* = 0.0812; sex × genotype interaction, *F*_(1, 54)_ = 0.01476, *P* = 0.9037. Correct day 2: sex factor, *F*_(1, 54)_ = 1.406, *P* = 0.2409; genotype factor, *F*_(1, 54)_ = 2.330, *P* = 0.1328; sex × genotype interaction, *F*_(1, 54)_ = 0.6338, *P* = 0.4294. We tested 18 female *App^h/h^*, 19 male *App^h/h^*, 10 female *App^s/s^*, and 11 male *App^s/s^*. No animals met the exclusion criteria of <25 visits per drinking session, and none died during the behavioral experiments.

Upon acquisition of spatial learning, reversal learning was tested in *App^h/h^* and *App^s/s^* rats using a modified place learning program, “Place Learning with Corner Switch,” wherein the assigned “correct” corner changes to an adjacent corner every 45 min during the testing window, over the course of two days (Figure 2A). As before, areas under the learning curve for each individual rat were derived and used to make comparisons between performance during the different days of the “Place Learning with Corner Switch” trial, and between sex-dependent and genotype-dependent effects on task performance (Figures 2B,C). There was no difference in task performance between the first and second day of the experiment for any of the groups tested (Figure 2B). Within each day and within the same genotype, task performance was not significantly different between sexes and genotypes (Figure 2C).

**FIGURE 3:**
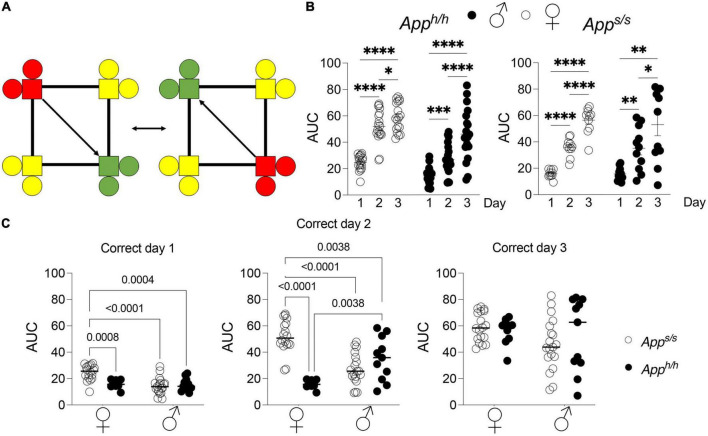
Behavioral sequencing analysis of *App^h/h^* and *App^s/s^* rats. **(A)** Schematic of behavioral sequencing program on top left, for a rat assigned to corner 2 or 4. Green indicates “correct” corner which then alternates to an adjacent corner after a nosepoke, with yellow indicating “incorrect” corners and red indicating “opposite i.e., incorrect, previously correct” corner. **(B)** Area under the “correct learning curve analyses for individual rats stratified by each day of the drinking session. Data are represented as mean ± SEM and were analyzed by two–way RM ANOVA followed by *post-hoc* Sidak’s multiple comparisons test when ANOVA showed significant differences (*App^h/h^* rats: day factor, *F*_(1.870, 65.45)_ = 118.6, *P* < 0.0001; sex factor, *F*_(1, 35)_ = 27.08, *P* < 0.0001; day × sex interaction, *F*_(2, 70)_ = 6.697, *P* = 0.0022; *post-hoc* Sidak’s multiple comparisons test, females day 1 vs. day 2 *P* < 0.0001; day 1 vs. day3 *P* < 0.0001; day 2 vs. day 3 *P* = 0.0359, males day 1 vs. day 2 *P* = 0.0006; day 1 vs. day3 *P* < 0.0001; day 2 vs. day 3 *P* < 0.0001. *App^s/s^* rats: day factor, *F*_(1.525, 28.98)_ = 65.85, *P* < 0.0001; sex factor, *F*_(1, 19)_ = 0.09169, *P* = 7653; day × sex interaction, *F*_(2, 38)_ = 0.1354, *P* = 0.8738; *post-hoc* Sidak’s multiple comparisons test, females day 1 vs. day 2 *P* < 0.0001; day 1 vs. day3 *P* < 0.0001; day 2 vs. day 3 *P* < 0.0001, males day 1 vs. day 2 *P* = 0.0016; day 1 vs. day3 *P* = 0.0012; day 2 vs. day 3 *P* = 0.0450). *p < 0.05, ^**^p < 0.01, ^***^p < 0.001, ^****^p < 0.0001. **(C)** Area under the “correct” learning curve analysis for individual rats stratified by sex and genotype. Data are represented as mean ± SEM and were analyzed by two–way ANOVA followed by *post-hoc* Sidak’s multiple comparisons test when ANOVA showed significant differences. Correct day 1: sex factor, *F*_(1, 54)_ = 12.30, *P* = 0.0009; genotype factor, *F*_(1, 54)_ = 6.694, *P* = 0.0124; sex × genotype interaction, *F*_(1, 54)_ = 11.39, *P* = 0.0014; *post-hoc* Sidak’s multiple comparisons test, female *App^h/h^* vs. female *App^s/s^ P* = 0.0008; female *App^h/h^* vs. male *App^h/h^ P* < 0.0001; female *App^h/h^* vs. male *App^s/s^ P* = 0.004; female *App^s/s.^* vs. male *App^h/h^ P* = 0.9882; female *App^s/s.^* vs. male *App^s/s^ P* > 0.9999; male *App^h/h^* vs. male *App*^s/s^* P* = 0.9939. Correct day 2: sex factor, *F*_(1, 54)_ = 0.7694, *P* = 0.3843; genotype factor, *F*_(1, 54)_ = 17.69, *P* < 0.0001; sex × genotype interaction, *F*_(1, 54)_ = 44.50, *P* < 0.0001; *post-hoc* Sidak’s multiple comparisons test, female *App^h/h^* vs. female *App^s/s^ P* < 0.0001; female *App^h/h^* vs. male *App^h/h^ P* < 0.0001; female *App^h/h^* vs. male *App^s/s^ P* = 0.0038; female *App^s/s.^* vs. male *App^h/h^ P* = 0.1334; female *App^s/s.^* vs. male *App^s/s^ P* = 0.0038; male *App^h/h^* vs. male *App*^s/s^* P* = 0.3970. Correct day 3: sex factor, *F*_(1, 54)_ = 2.865, *P* = 0.0963; genotype factor, *F*_(1, 54)_ = 0.1859, *P* = 0.681; sex × genotype interaction, *F*_(1, 54)_ = 0.9552, *P* = 0.3327. We tested 18 female *App^h/h^*, 19 male *App^h/h^*, 10 female *App^s/s^*, and 11 male *App^s/s^*. No animals met the exclusion criteria of <25 visits per drinking session, and none died during the behavioral experiments.

Next, rats were analyzed with the “Behavioral Sequencing” test. In this task, each rat is again assigned a “correct” corner, but a visit to the “correct” corner then triggers the reassignment of the “correct” corner to the opposite corner (Figure 3A). Rats must find their “correct” corner, and then learn to shuttle back and forth between diagonally opposing corners for water access, while avoiding the never-rewarded “lateral” corners. This test was modeled on the Brixton Spatial Anticipation test, which measures spatial discrimination and flexibility and can reveal deficits in higher order brain function (Almkvist and Winblad, 1999). This test has been developed for the IntelliCage previously and found to accurately measure executive function across different mouse species with high inter-laboratory reproducibility.

For quantitative analysis of task performance in “Behavioral Sequencing,” the area under the curve of each individual rat was calculated. Comparisons were then made between performances during the different days of the “Behavioral Sequencing” trial, and between sex-dependent and genotype-dependent effects on task performance (Figures 3B,C). Within each genotype and sex, there were statistically significant increases in task performance across every day for each group, as measured by area under the “correct” learning curve (Figure 3B). Genotype and sex-dependent deficits in task performance were detected. On days 1 and 2 of the “Behavioral Sequencing” task, female *App^h/h^* rats performed significantly better when compared to female *App^s/s^*, male *App^s/s^* and male *App^h/h^* rats, though by day 3 the performances of the four groups were comparable (Figure 3C). On days 2, female *App^s/s^* animals performed significantly worse than male *App^s/s^* rats as well (Figure 3C). In summary, genotype-dependent differences in task performance were present in female rats, with *App^s/s^* rats performing significantly worse as compared to female *App^h/h^* animals, but not in male *App^s/s^* and *App^h/h^* rats. In addition, sex-dependent differences were identified, with female *App^h/h^* rats performing significantly better as compared to male *App^h/h^* and *App^s/s^* rats.

### Male and female *App^s/s^* rats exhibit increased Aβ40 and Aβ42 levels without simultaneous changes in Aβ oligomers and Alzheimer’s disease brain amyloid pathology

**FIGURE 4:**
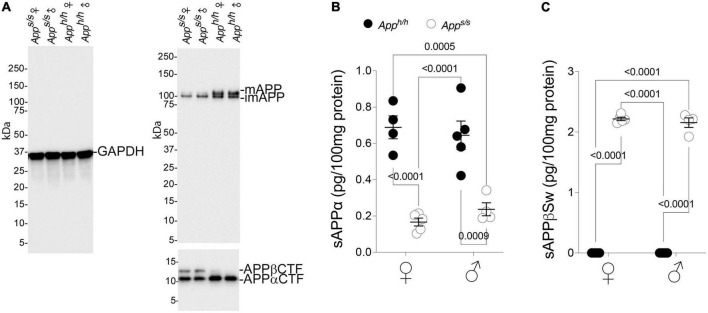
Increased processing by β-secretase and decreased processing by α-secretase of Swedish APP. **(A)** Western blot (WB) of brain lysates from *App^s/s^* and *App^h/h^* rats with an anti-GAPDH antibody (left) and the anti C-terminal APP antibody Y188 (right). A longer exposure was used to visualize the two C-Terminal APP fragments βCTF and αCTF (bottom). The samples analyze contain equal amounts of brain lysates from: *App^h/h^* females *n* = 4, *App^s/s^* females *n* = 5, *App^h/h^* males *n* = 5, *App^s/s^* males *n* = 4. **(B)** Levels of sAPPα were determined by ELISA (*App^h/h^* females *n* = 4, *App^s/s^* females *n* = 5, *App^h/h^* males *n* = 5, *App^s/s^* males *n* = 4). Data are represented as mean ± SEM and were analyzed by two–way ANOVA followed by *post-hoc* Sidak’s multiple comparisons test when ANOVA showed significant differences (sex factor, *F*_(1, 14)_ = 0.05753, *P* = 0.8139; genotype factor, *F*_(1, 14)_ = 69.01, *P* < 0.0001; sex × genotype interaction, *F*_(1, 14)_ = 1.037, *P* = 0.3259; *post-hoc* Sidak’s multiple comparisons test, female *App^h/h^* vs. female *App^s/s^ P* < 0.0001; female *App^h/h^* vs. male *App^h/h^ P* = 0.9953; female *App^h/h^* vs. male *App^s/s^ P* = 0.0005; female *App^s/s.^* vs. male *App^h/h^ P* < 0.0001; female *App^s/s.^* vs. male *App^s/s^ P* = 0.9478; male *App^h/h^* vs. male *App*^s/s^* P* = 0.0009). **(C)** Levels of sAPPβSw were determined by ELISA (*App^h/h^* females *n* = 4, *App^s/s^* females *n* = 5, *App^h/h^* males *n* = 5, *App^s/s^* males *n* = 4). Control samples have no signal because the ELISA does not recognize wild type sAPPβ. Data are represented as mean ± SEM and were analyzed by two–way ANOVA followed by *post-hoc* Sidak’s multiple comparisons test when ANOVA showed significant differences (sex factor, *F*_(1, 14)_ = 0.6506, *P* = 0.4334; genotype factor, *F*_(1, 14)_ = 3347, *P* < 0.0001; sex × genotype interaction, *F*_(1, 14)_ = 0.6531, *P* = 0.4325; *post-hoc* Sidak’s multiple comparisons test, female *App^h/h^* vs. female *App^s/s^ P* < 0.0001; female *App^h/h^* vs. male *App^h/h^ P* > 0.9999; female *App^h/h^* vs. male *App^s/s^ P* < 0.0001; female *App^s/s.^* vs. male *App^h/h^ P* < 0.0001; female *App^s/s.^* vs. male *App^s/s^ P* = 0.8520; male *App^h/h^* vs. male *App*^s/s^* P* < 0.0001).

APP is cleaved by several proteases: the most studied pathways involve cleavage by α-, β- and γ-secretases. Cleavage of APP by the β-secretase releases the soluble APP ectodomain sAPPβ, and the membrane-bound fragment βCTF. βCTF is cleaved with lax site specificity by γ-secretase into Aβ peptides of different lengths and the APP intracellular domain (AID). Alternatively, α-secretase cleaves APP within the Aβ sequence to produce sAPPα and αCTF. αCTF can be also cleaved by γ-secretase into a “shorter Aβ” peptide, called P3, and AID. The human APP Swedish mutation leads to increased β-secretase processing of APP (Citron et al., 1992, 1994; Johnston et al., 1994). Analysis of young *App^s/s^* rats showed that this biochemical change is reproduced in this knock-in rat line (Tambini et al., 2020). In addition, we described a reduction in mature APP levels and in the rate of APP cleavage by α-secretase in young *App^s/s^* rats (Tambini et al., 2020). To determine whether these metabolic changes are also evident in older *App^s/s^* rats and whether they associated with behavioral performance, some of the rats tested in behavior were used to study APP metabolism. Qualitative western blot analysis of representative lysates mixes, in which a mixture of equal amounts of brain lysates from 4 female *App*^h/h^**, 5 male *App^h/h^*, 5 female *App^s/s^* and 4 male *App^s/s^* rats were run as pooled samples for each sex/genotype, shows an increase in βCTF levels and a decrease in αCTF and mature APP levels in *App^s/s^* rats compared to control *App^h/h^* animals for both sexes (Figure 4A). Quantitative ELISA shows that sAPPα levels were reduced in 14 months old male and female *App^s/s^* rats (Figure 4B), confirming the changes observed young *App* Swedish rats (Tambini et al., 2020). Finally, an ELISA specific for Swedish mutant sAPPβ shows that levels of sAPPβSw are similar in male and female *App^s/s^* rats (Figure 4C).

**FIGURE 5:**
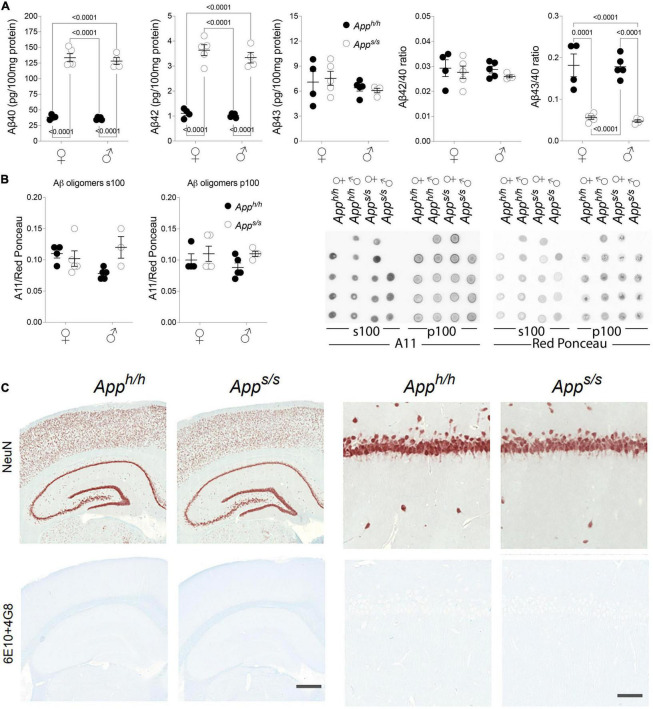
Analysis of Aβ species and amyloid pathology in *App^h/h^* and *App^s/s^* rats brains. **(A)** ELISA levels of Aβ40, Aβ42 and Aβ43 in *App^h/h^* females *n* = 4, *App^s/s^* females *n* = 5, *App^h/h^* males *n* = 5, *App^s/s^* males *n* = 4. Ratio of Aβ42/Aβ40 and Aβ43/Aβ40 are also presented. Data are represented as mean ± SEM and were analyzed by two–way ANOVA followed by *post-hoc* Sidak’s multiple comparisons test when ANOVA showed significant differences (Aβ40: sex factor, *F*_(1, 14)_ = 0.7569, *P* = 0.3990; genotype factor, *F*_(1, 14)_ = 440.9, *P* < 0.0001; sex × genotype interaction, *F*_(1, 14)_ = 0.1036, *P* = 0.7523; *post-hoc* Sidak’s multiple comparisons test, female *App^h/h^* vs. female *App^s/s^ P* < 0.0001; female *App^h/h^* vs. male *App^h/h^ P* = 0.9993; female *App^h/h^* vs. male *App^s/s^ P* < 0.0001; female *App^s/s.^* vs. male *App^h/h^ P* < 0.0001; female *App^s/s.^* vs. male *App^s/s^ P* = 0.9593; male *App^h/h^* vs. male *App*^s/s^* P* < 0.0001. Aβ42: sex factor, *F*_(1, 14)_ = 1.379, *P* = 0.2598; genotype factor, *F*_(1, 14)_ = 224.3, *P* < 0.0001; sex × genotype interaction, *F*_(1, 14)_ = 0.4704, *P* = 0.5040; *post-hoc* Sidak’s multiple comparisons test, female *App^h/h^* vs. female *App^s/s^ P* < 0.0001; female *App^h/h^* vs. male *App^h/h^ P* = 0.9997; female *App^h/h^* vs. male *App^s/s^ P* < 0.0001; female *App^s/s.^* vs. male *App^h/h^ P* < 0.0001; female *App^s/s.^* vs. male *App^s/s^ P* = 0.7560; male *App^h/h^* vs. male *App*^s/s^* P* < 0.0001. Aβ43: sex factor, *F*_(1, 14)_ = 1.877, *P* = 0.1923; genotype factor, *F*_(1, 14)_ = 0.006110, *P* = 0.9388; sex × genotype interaction, *F*_(1, 14)_ = 0.2458, *P* = 0.6277. Aβ42/Aβ40: sex factor, *F*_(1, 14)_ = 0.2542, *P* = 0.6220; genotype factor, *F*_(1, 14)_ = 1.009, *P* = 0.3322; sex × genotype interaction, *F*_(1, 14)_ = 0.06414, *P* = 0.8037. Aβ40/Aβ43: sex factor, *F*_(1, 14)_ = 0.1730, *P* = 0.6838; genotype factor, *F*_(1, 14)_ = 83.80, *P* < 0.0001; sex × genotype interaction, *F*_(1, 14)_ = 0.03059, *P* = 0.8637; *post-hoc* Sidak’s multiple comparisons test, female *App^h/h^* vs. female *App^s/s^ P* = 0.0001; female *App^h/h^* vs. male *App^h/h^ P* > 0.9999; female *App^h/h^* vs. male *App^s/s^ P* < 0.0001; female *App^s/s.^* vs. male *App^h/h^ P* < 0.0001; female *App^s/s.^* vs. male *App^s/s^ P* = 0.9990; male *App^h/h^* vs. male *App*^s/s^* P* < 0.0001). **(B)** Quantitation of oligomeric Aβ detected by dot-blots using the oligomer-specific antibody A11. Both the s100 and p100 brain fractions were tested. Before immunoblot analysis, membranes were stained with Ponceau red. Quantitative analysis of A11 blot was normalized to the Ponceau red quantitative analysis. We analyzed: *App^h/h^* females *n* = 4, *App^s/s^* females *n* = 5, *App^h/h^* males *n* = 5, *App^s/s^* males *n* = 4. However, the s100 fraction of one *App^s/s^* male sample, which gave a red Ponceau signal but not an A11 signal, was excluded. Data are represented as mean ± SEM and were analyzed by two–way ANOVA followed by *post-hoc* Sidak’s multiple comparisons test when ANOVA showed significant differences (oligomeric Aβ in s100: sex factor, *F*_(1, 13)_ = 0.4499, *P* = 0.5141; genotype factor, *F*_(1, 13)_ = 2.6523, *P* = 0.1273; sex × genotype interaction, *F*_(1, 13)_ = 2.738, *P* = 0.1591. Oligomeric Aβ in p100: sex factor, *F*_(1, 14)_ = 0.4088, *P* = 0.5329; genotype factor, *F*_(1, 14)_ = 2.907, *P* = 0.1103; sex × genotype interaction, *F*_(1, 14)_ = 0.4088, *P* = 0.5329). The dot blot images (WB with A11 and Red Ponceau staining) are shown on the right. **(C)** Histopathological analysis of 14-month-old *App^h/h^* and *App^s/s^* rats (*App^h/h^*, 5 male and 4 female rats, and *App^s/s^*, 5 male and 5 female). The left panels show representative images of the anterior hippocampus and overlaying somatosensory cortex of *App^h/h^* and *App^s/s^* rat brains. Illustrates of, from the top to bottom, neurons (NeuN) and Amyloidβ (6E10+4G8). The scale bar is equivalent to 500 microns. The right panels show high-magnification picture of the hippocampal CA1 subregion for the staining depicted in the left panels. The scale bar is equivalent to 50 microns.

Increased rate of APP cleavage by β-secretase results in increased Aβ production in young *App^s/s^* rats. The longer forms of Aβ, such as Aβ42 and Aβ43, are considered the neurotoxic APP metabolites responsible for AD pathogenesis, cognitive impairments and neurodegeneration. Thus, we measured Aβ levels in the brains of these 14 months old rats and found that *App^s/s^* rats produce significantly more human Aβ40 and Aβ42 as compared to control animals; additionally, Aβ40 and Aβ42 increased equivalently in male and female *App^s/s^* rats (Figure 5A). Surprisingly perhaps, Aβ43 levels were similar in all 4 rat groups (Figure 5A).

Increases in the ratios of long pathogenic (Aβ42 and Aβ43) over short not pathogenic Aβ species (Aβ40) are also believed to trigger AD. Yet, the Aβ42/Aβ40 ratio was comparable in *App^s/s^* rats and control *App^h/h^* animals of both sexes, and the Aβ43/Aβ40 ratio was reduced in both male and female *App^s/s^* rats compared to male and female *App^h/h^* animals (Figure 5A).

It has been postulated that toxic forms of Aβ are oligomeric (Shankar et al., 2008). Thus, we tested whether toxic oligomers are augmented in *App^s/s^* rats, and whether this augmentation is sex specific. To this end, we used the prefibrillar oligomer-specific antibody A11 to perform dot blots (Kayed et al., 2003). We found no evidence supporting an increase in neurotoxic brain oligomer levels in *App^s/s^* rats of either sex as compared to male and female *App^h/h^* control rats (Figure 5B).

Aβ may exert neurotoxic effect by depositing in pathological lesions know as amyloid plaques. According to this hypothesis, Aβ is accumulated and aggregated or oligomerized to form amyloid plaques, which subsequently activates disease associated microglia and astrocytes, formed neurofibrillary tangles (NFT) and finally leads to neuronal loss or dysfunction and dementia. Amyloid pathology was absent in young *App^s/s^* rats, despite an increase in Aβ (Tambini et al., 2019). To determine whether Amyloid pathology was present in older *App^s/s^* rats, and whether this pathology is sex specific, we used immunohistochemistry (IHC) analysis to characterize brains from some of the rats tested in behavioral tasks. Regions of analysis included the frontal cortex, cingulate cortex, whole hippocampus, and entorhinal cortex. IHC staining was performed on *App^h/h^* (5 male and 4 female rats), and *App^s/s^* (5 male and 5 female rats). No observable differences in gross brain morphology or cellularity were observed and comparison between males and females did not reveal any sex-related differences (Figure 5C). The 6E10+4G8 antibodies targeting respectively amino acids 1–17 and 18–23 of the APP Aβ region, were mixed and used to investigate the presence of amyloid plaque. Amyloid plaques were not detected by IHC in any of the animals studied (Figure 5C). In summary, we could not describe alterations in Aβ metabolism that, per se’, are associated with the behavioral phenotype.

### Male and female *App^s/s^* rats show no evidence of tau hyperphosphorylation, tau pathology, and neuroinflammation

**FIGURE 6:**
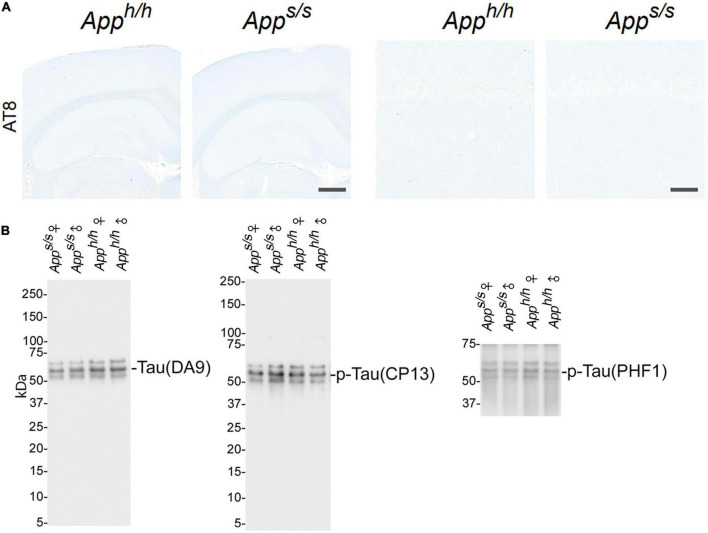
Analysis of tau pathology and phosphorylation in *App^h/h^* and *App^s/s^* rats brains. **(A)** Histopathological analysis, using the anti-phospho-Tau antibody AT8 staining, of 14-month-old *App^h/h^* and *App^s/s^* rats (*App^h/h^*, 5 male and 4 female rats, and *App^s/s^*, 5 male and 5 female). The left panels show representative images of the anterior hippocampus and overlaying somatosensory cortex of *App^h/h^* and *App^s/s^* rat brains. The scale bar is equivalent to 500 microns. The right panels show high-magnification picture of the hippocampal CA1 subregion for the staining depicted in the left panels. The scale bar is equivalent to 50 microns. **(B)** WB of brain lysates from *App^s/s^* and *App^h/h^* rats with DA9, CP13 and PHF1, which recognize: total Tau, tau phosphorylated on S^202^, and tau phosphorylated on S^396–404^, respectively. The samples analyze are the same used in Figure 4A.

As discussed above, NFTs are another characteristic neuropathological lesion of AD. NFTs are composed of highly phosphorylated forms of the microtubule-associated protein tau. Tau phosphorylation was evaluated with the AT8 antibody. NFTs and p-tau were not detected by IHC in any of the animals studied (Figure 6A). To further test tau phosphorylation status, representative lysates mixes (see Figure 4A) were analyzed by western blot using the anti tau mouse monoclonal antibodies DA9, CP13 and PHF1, which recognize: total Tau, tau phosphorylated on S^202^, and tau phosphorylated on S^396–404^, respectively. This analysis showed no obvious differences in total tau and p-tau among the four rat groups (Figure 6B).

**FIGURE 7:**
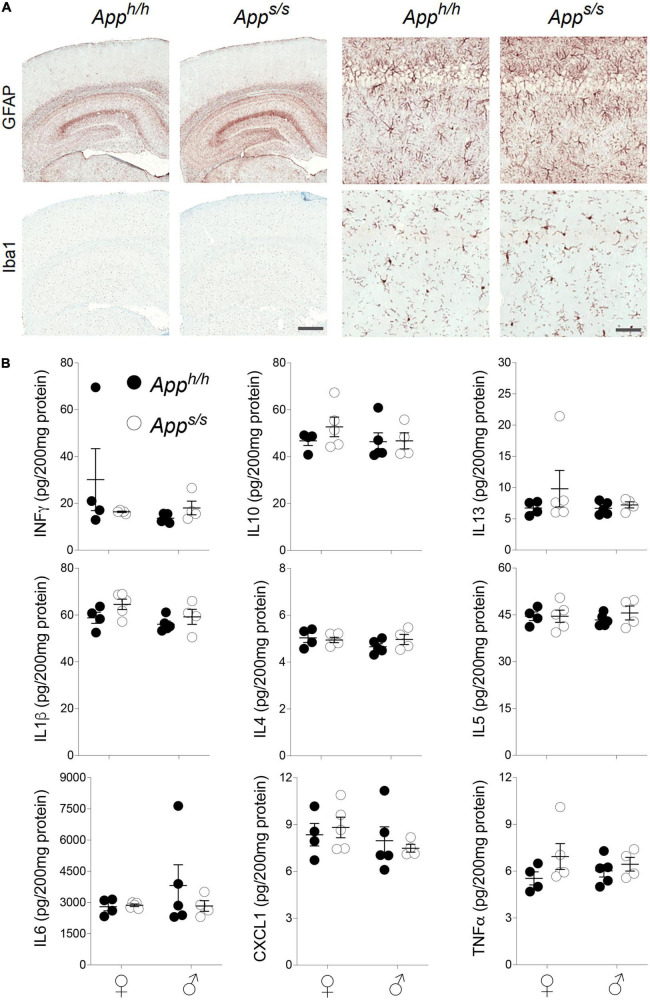
Neuroinflammation analysis in *App^h/h^* and *App^s/s^* rats. **(A)** Histopathological analysis, using the astrocytes (GFAP) and microglia (Iba1) markers, of 14-month-old *App^h/h^* and *App^s/s^* rats (*App^h/h^*, 5 male and 4 female rats, and *App^s/s^*, 5 male and 5 female). The left panels show representative images of the anterior hippocampus and overlaying somatosensory cortex of *App^h/h^* and *App^s/s^* rat brains. The scale bar is equivalent to 500 microns. The right panels show high-magnification picture of the hippocampal CA1 subregion for the staining depicted in the left panels. The scale bar is equivalent to 50 microns. **(B)** Levels of IFN-δ, IL-1β, IL-4, IL-5, IL-6, CXCL1, IL-10, IL-13, and TNF-α in the CNS of 14 months old *App^h/h^* and *App^s/s^* rats (*App^h/h^*, 5 male and 4 female rats, and *App^s/s^*, 4 male and 5 female) were measured by ELISA. Data are represented as mean ± SEM and were analyzed by two–way ANOVA followed by *post-hoc* Sidak’s multiple comparisons test when ANOVA showed significant differences (IFN-γ: sex factor, *F*_(1, 14)_ = 1.541, *P* = 0.2348; genotype factor, *F*_(1, 14)_ = 0.6209, *P* = 0.4438; sex × genotype interaction, *F*_(1, 14)_ = 2.295, *P* = 0.1520. IL-10: sex factor, *F*_(1, 14)_ = 0.7531, *P* = 0.4001; genotype factor, *F*_(1, 14)_ = 0.7504, *P* = 0.4010; sex × genotype interaction, *F*_(1, 14)_ = 0.5898, *P* = 0.4553. IL-13: sex factor, *F*_(1, 14)_ = 0.5895, *P* = 0.4554; genotype factor, *F*_(1, 14)_ = 1.128, *P* = 0.3062; sex × genotype interaction, *F*_(1, 14)_ = 0.5577, *P* = 0.4675. IL-1β: sex factor, *F*_(1, 14)_ = 3.074, *P* = 0.1014; genotype factor, *F*_(1, 14)_ = 3.743, *P* = 0.0735; sex × genotype interaction, *F*_(1, 14)_ = 0.3192, *P* = 0.5810. IL-4: sex factor, *F*_(1, 14)_ = 1.239, *P* = 0.2844; genotype factor, *F*_(1, 14)_ = 0.4730, *P* = 0.5029; sex × genotype interaction, *F*_(1, 14)_ = 1.550, *P* = 0.2336. IL-5: sex factor, *F*_(1, 14)_ = 0.001888, *P* = 0.9660; genotype factor, *F*_(1, 14)_ = 0.4417, *P* = 0.5171; sex × genotype interaction, *F*_(1, 14)_ = 0.4502, *P* = 0.5131. IL-6: sex factor, *F*_(1, 14)_ = 0.7154, *P* = 0.4119; genotype factor, *F*_(1, 14)_ = 0.6159, *P* = 0.4457; sex × genotype interaction, *F*_(1, 14)_ = 0.8091, *P* = 0.3836. CXCL1: sex factor, *F*_(1, 14)_ = 1.453, *P* = 0.2481; genotype factor, *F*_(1, 14)_ = 0.0001067, *P* = 0.9919; sex × genotype interaction, *F*_(1, 14)_ = 0.4418, *P* = 0.5150. TNF-α: sex factor, *F*_(1, 14)_ = 0.00004924, *P* = 0.9945; genotype factor, *F*_(1, 14)_ = 2.434, *P* = 0.1411; sex × genotype interaction, *F*_(1, 14)_ = 0.7000, *P* = 0.4160).

A pathogenic role for neuroinflammation in AD has emerged (Akiyama et al., 2000; Tarkowski et al., 2003). Variants of *TREM2*, whose CNS expression is restricted to microglia (Schmid et al., 2002), increase the risk of AD (Guerreiro et al., 2013), consolidating the neuroinflammation-AD link. Thus, we tested whether *App^s/s^* rats showed signs of neuroinflammation. First, we used the astrocytes (GFAP) and microglia (Iba1) markers in IHC analysis. The staining intensity and morphology of microglia and astrocytes were similar across the two genotypes and sexes (Figure 7A). Next, we used an ELISA multiplex to quantify 9 cytokines (IFN-γ, IL-1β, IL-4, IL-5, IL-6, CXCL1, IL-10, IL-13, and TNF-α) that are important in inflammation response and immune system regulation. We measured cytokine levels in the CNS of the 14 months-old rats tested for Aβ levels (Figure 5). Levels of all 9 cytokines were not significantly different in the four rat groups analyzed (Figure 7B). In summary, the data suggest that, at 14 months of age, the Swedish APP mutation does not trigger obvious tau-pathology and neuroinflammation.

### Long-term potentiation is impaired at hippocampal Schaffer collateral-CA1 synapses of 12 months old *App^s/s^* rats

**FIGURE 8:**
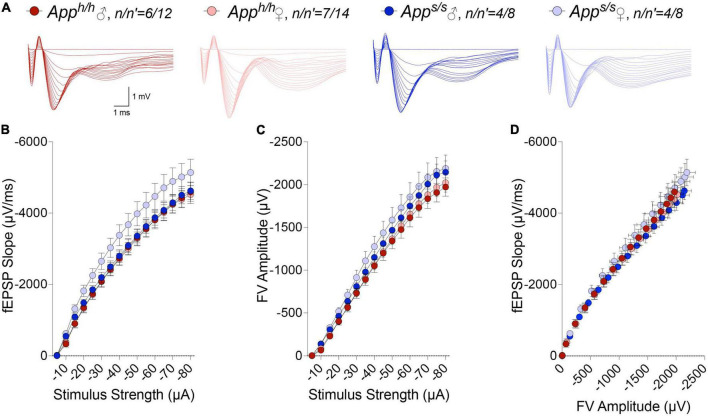
I/O responses analysis in *App^h/h^* and *App^s/s^* rats. **(A)** Representative traces of fEPSP in response to increasing stimulus from -5 to -80 μA. **(B)** I-O curve generated from the slope fEPSP versus stimulus strength (2-way ANOVA summary, *F*_(3, 38)_ = 1.710; *p* = 0.1813). **(C)** I-O curve generated from FV amplitude versus stimulus strength (2-way ANOVA summary, *F*_(3, 38)_ = 1.262, *p* = 0.3010). **(D)** I-O curve generated from the slope fEPSP versus FV amplitude. Data are represented as mean ± SEM. Data were analyzed by two-way ANOVA for repeated measures followed by *post-hoc* Tukey’s multiple comparisons test when ANOVA showed statistically significant differences. *n* = number of animals; *n*′ = number of slices.

**FIGURE 9:**
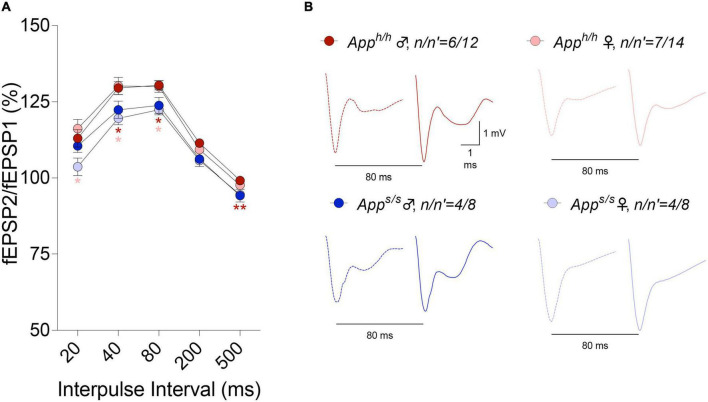
Analysis of PPR in *App^h/h^* and *App^s/s^* rats. **(A)** In *App^s/s^* rats, PPR were decreased at 500, 200, 80, and 40 ms IPI compared to control male rats and at 80, 40, and 20 ms IPI compared to control female rats (2-way ANOVA summary, *F*_(3, 38)_ = 4.573, *p* = 0.0079; *post-hoc* Tukey’s multiple comparison test: at 500 ms IPI *App^s/s^* female vs *App^h/h^* male *p* = 0.0061**, *App^s/s^* female vs *App^s/s^* male *p* = 0.9991, *App^s/s^* female vs *App^h/h^* female *p* = 0.0798, *App^h/h^* female vs *App^s/s^* male *p* = 0.4504, *App^h/h^* male vs *App^s/s^* male *p* = 0.1825, *App^h/h^* male vs *App^h/h^* female *p* = 0.3847; at 200 ms IPI *App^s/s^* female vs *App^h/h^* male *p* = 0.0055**, *App^s/s^* female vs *App^s/s^* male *p* = 0.9982, *App^s/s^* female vs *App^h/h^* female *p* = 0.0840, *App^h/h^* female vs *App^s/s^* male *p* = 0.6325, *App^h/h^* male vs *App^s/s^* male *p* = 0.2409, *App^h/h^* male vs *App^h/h^* female *p* = 0.0852; at 80 ms IPI *App^s/s^* female vs *App^h/h^* male *p* = 0.0137*, *App^s/s^* female vs *App^h/h^* female *p* = 0.0364*, *App^s/s^* female vs *App^s/s^* male *p* = 0.9683, *App^h/h^* female vs *App^s/s^* male *p* = 0.2707, *App^h/h^* male vs *App^s/s^* male *p* = 0.1872, *App^h/h^* male vs *App^h/h^* female *p* = 0.9982; at 40 ms IPI *App^s/s^* female vs *App^h/h^* male *p* = 0.0203*, *App^s/s^* female vs *App^h/h^* female *p* = 0.0317*, *App^s/s^* female vs *App^s/s^* male *p* = 0.8634, *App^h/h^* female vs *App^s/s^* male *p* = 0.2400, *App^h/h^* male vs *App^s/s^* male *p* = 0.2340, *App^h/h^* male vs *App^h/h^* female *p* = 0.9971; at 20 ms IPI *App^s/s^* female vs *App^h/h^* male *p* = 0.1407, *App^s/s^* female vs *App^h/h^* female *p* = 0.0345*, *App^s/s^* female vs *App^s/s^* male *p* = 0.2703, *App^h/h^* female vs *App^s/s^* male *p* = 0.4372, *App^h/h^* male vs *App^s/s^* male *p* = 0.8996, *App^h/h^* male vs *App^h/h^* female *p* = 0.8708). **(B)** Representative traces of fEPSP evoked at 80 ms IPI are shown. Dotted lines represent the response after the first stimulation and solid lines represent the second responses. Data are represented as mean ± SEM. Data were analyzed by two-way ANOVA for repeated measures followed by *post-hoc* Tukey’s multiple comparisons test when ANOVA showed statistically significant differences. *n* = number of animals; *n*′ = number of slices.

LTP, a long-lasting form of synaptic plasticity, has been described at glutamatergic synapses throughout the brain and remains one of the most attractive cellular models for learning and memory. Given that increased β-secretase processing of the Swedish APP mutant facilitates glutamate release in *App^s/s^* rats, *via* a mechanism defined as β-secretase and APP−dependent glutamate release (BAD−Glu) (Tambini et al., 2019), we determined whether the *App^s^* variant could also impact this electrophysiological surrogate of memory in 12 months old animals. Behavioral sequencing task is based on Brixton Spatial Anticipation task which is used for the clinical assessment of human cognitive functions via visuospatial sequencing task (Endo et al., 2011). The hippocampus has important roles both in visuospatial memory and episodic memory (Smith and Milner, 1981; Squire and Zola-Morgan, 1991; Eichenbaum, 2017). In addition, hippocampal but not striatal lesions impaired learning in Intellicage behavioral tasks (Voikar et al., 2010). Thus, we carried out all electrophysiology recording in hippocampal Schaffer Collateral-CA1 synapses. Before recording LTP, basal synaptic transmission (BST) at the hippocampal Schaffer Collateral-CA1 synapses was examined using the slope of field excitatory postsynaptic potentiation (fEPSP) evoked by increasing current stimulation. BST was similar in all 4 animal groups (representative traces are shown in Figure 8A, data are shown in Figure 8B). The amplitudes of fiber volley (FV), an indicator of the size of the ascending fiber stimulus, were also measured showing no genotype/sex-dependent differences (Figure 8C). We also examined FV amplitudes versus evoked fEPSP slope and I/O curves were indistinguishable in hippocampal slices from *App^s/s^* and *App^h/h^* rats (Figure 8D). We next analyzed paired-pulse responses (PPR), a form of short-term synaptic plasticity that is determined, at least in part, by changes in release Probability (P*r*) of glutamatergic synaptic vesicles; an increase in P*r* leads to a decrease in facilitation (Zucker and Regehr, 2002). PPR recordings were performed using a stimulation intensity that elicited a response 40% of the maximum evoked response in BST recordings. To induce PPR, two stimuli were applied at either 20, 40, 80, 200, or 500 ms intervals. A significantly lower PPR was observed in *App^s/s^* female rats at inter-pulse-interval (IPI) of 500, 80, 40 ms compared to *App^h/h^* male rats and at IPI of 80, 40, 20 ms compared to *App^h/h^* female rats (Figures 9A,B). The PPR of *App^s/s^* male rats was also lower compared to male and female *App^h/h^* animals, albeit these differences did not reach statistical significance.

**FIGURE 10:**
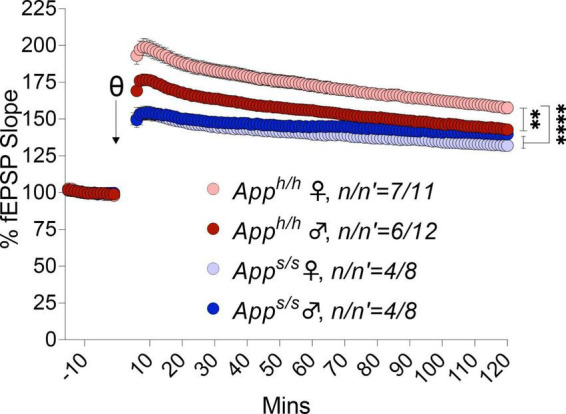
LTP analysis in *App^h/h^* and *App^s/s^* rats. LTP recording in both genders of *App^s/s^* are weaker than controls. Additionally, in male *App^h/h^* is weaker compared to female *App^h/h^* (2-way ANOVA summary: female *App^h/h^* vs. female *App^s/s^ F*_(1, 17)_ = 36.82, *p* < 0.0001****; female *App^h/h^* vs. male *App^s/s^ F*_(1, 17)_ = 25.48, *p* < 0.0001****; female *App^h/h^* vs. male *App^h/h^ F*_(1, 21)_ = 11.54, *p* = 0.0027**; female *App^s/s^* vs. male *App^s/s^ F*_(1, 14)_ = 1.266, *p* = 0.2795; male *App^h/h^* vs. male *App^s/s^ F*_(1, 18)_ = 5.355, *p* = 0.0327*; male *App^h/h^* vs. female *App^s/s^ F*_(1, 18)_ = 12.02, *p* = 0.0028**). Each genotype/gender were compared separately. Data are represented as mean ± SEM. Data were analyzed by two-way ANOVA (Column factor). *n* = number of animals; *n*′ = number of slices.

**FIGURE 11:**
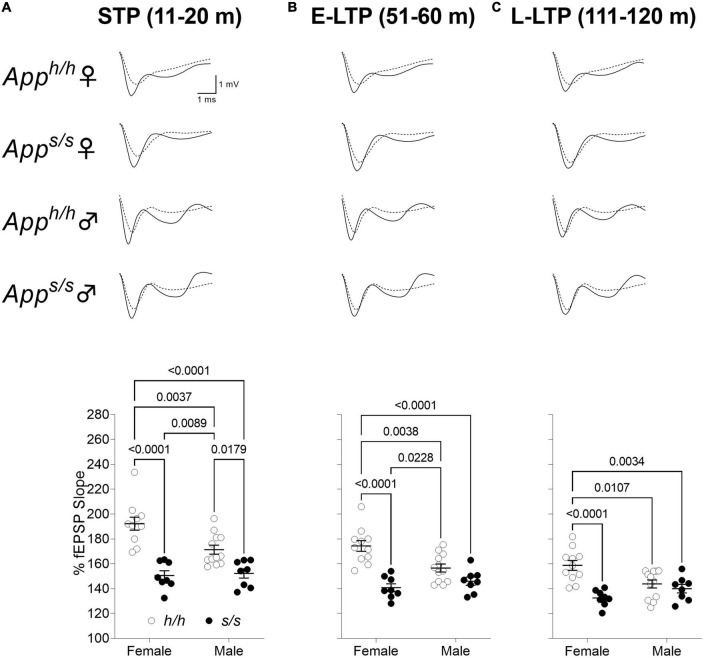
Swedish mutation impaired all phases of LTP. **(A)** Plot of fEPSP slope change in 11–20 m (short term potentiation, STP). The average traces of the baseline (dotted line) and STP (solid line) are shown on top. (ANOVA summary; sex factor, *F*_(1, 35)_ = 4.820, *p* = 0.0349; genotype factor, *F*_(1, 35)_ = 48.38 *p* < 0.0001; sex × genotype interaction, *F*_(1, 35)_ = 6.638, *p* = 0.0144). **(B)** Plot of fEPSP slope change in 51–60 m (early LTP, E-LTP). The average traces of the baseline (dotted line) and E-LTP (solid line) are shown on top. (ANOVA summary, sex factor, *F*_(1, 35)_ = 2.850, *p* = 0.1002; genotype factor, *F*_(1, 35)_ = 35.18 *p* < 0.0001; sex × genotype interaction, *F*_(1, 35)_ = 9.448, *p* = 0.0041). **(C)** Plot of fEPSP slope changes in 111–120 m (late-LTP, L-LTP). The average traces of the baseline (dotted line) and L-LTP (solid line) are shown on top. (ANOVA summary, sex factor, *F*_(1, 35)_ = 1.134, *p* = 0.2942; genotype factor, *F*_(1, 35)_ = 18.46 *p* = 0.0001; sex × genotype interaction, *F*_(1, 35)_ = 10.23, *p* = 0.0029). Data are represented as mean ± SEM. Data were analyzed by one-way ANOVA for repeated measures followed by *post-hoc* Tukey’s multiple comparisons test when ANOVA showed statistically significant differences.

We next examined LTP elicited by ⊖-burst stimulation (TBS). Before recording LTP, baseline was recorded every minute at an intensity that elicited a response 40% of the maximum evoked response. We observed a reduction in the analysis of whole LTP recording in both gender of *App^s/s^* rats compared to *App^h/h^* rats and in male *App^h/h^* compared to female *App^h/h^* rats (Figure 10). We then analyzed LTP as 3 different phases: short term potentiation (STP, 11–20 m), early-LTP (E-LTP, 51–60 m) and late-LTP (L-LTP, 111–120 m). The first phase of the LTP, STP, is dependent on N-methyl-D-aspartate (NMDA) receptors while E-LTP and L-LTP are related to protein kinase and protein synthesis, respectively (Huang and Kandel, 1994; Lauri et al., 2007). STP was reduced in both sexes in *App^s/s^* rats (Figure 11A). E-LTP was reduced in both *App^s/s^* sexes and also there is a reduction in *App^h/h^* males compared to *App^h/h^* females (Figure 11B). L-LTP was reduced in *App^s/s^* rats and in *App^h/h^* male rats compared to female *App^h/h^* (Figure 11C). Remarkably, the L-LTP profile parallels the behavioral sequencing task performances shown in Figure 3.

## Discussion

To determine whether Swedish *App* leads to cognitive deficits, and to assess whether sex is a biological variable affecting cognitive performances, *App^s/s^* knock-in and control *App^h/h^* knock-in male and female rats were subjected to behavioral analysis using the IntelliCage platform, an automated behavioral testing system that allows behavioral assessment in socially housed animals reflecting a more natural and less stress-inducing environment. With regards to genotype-dependent and sex-dependent differences in task performance, we see no differences in “Place Learning” and “Place Learning with Corner Switch” tasks (Figures 1, 2). Previously, in 4–5-month-old *App^h/h^* rats, we reported (Pham et al., 2022) that males tended to perform better than females in “Place Learning with Corner Switch”. By 13 months of age, as reported here, this difference is no longer apparent, and indeed the worsening relative trajectory of the male rats as a function of time may corroborate the finding that in 1-year-old mice, females outperformed males in IntelliCage place-learning behavioral experiments (Mifflin et al., 2021).

From the early phases of AD named mild cognitive impairment (MCI), episodic memory and visuospatial impairments are observed (Almkvist and Winblad, 1999). In this study, sex-dependent cognitive performance difference becomes apparent in “Behavioral sequencing” task of IntelliCage experiments. Behavioral sequencing task is based on Brixton Spatial Anticipation task which is used for the clinical assessment of human cognitive functions via visuospatial sequencing task (Endo et al., 2011). This task could also address behavioral flexibility in addition to spatial and temporal learning related to episodic memory. Here, we show a clear finding of improved task performance of females as compared to males within the *App^h/h^* genotype (Figure 3C), and in the Apps/s group, with Apps/s females performing significantly worse compared to male *App^s/^*on day 2 (Figure 3C). Our results also showed a genotype-dependent effect in females, with female *App^s/s^* rats performing significantly worse than control *App^h/h^* females (Figure 3C). Interestingly, a genotype-dependent effect was not detected in the male groups, with male *App^h/h^* and *App^s/s^* rats showing similar performance (Figure 3C). Overall, the data show that the interaction between the Swedish APP mutation and sex impairs episodic-like memory in rats similar to prodromal phase of AD.

The longer forms of Aβ, such as Aβ42 and Aβ43, are considered to be the neurotoxic APP metabolites responsible for AD pathogenesis, cognitive impairments, and neurodegeneration. An absolute increase in Aβ levels and/or increases in the ratios of long pathogenic (Aβ42 and Aβ43) over short Aβ species (Aβ40) are believed to trigger AD. Moreover, it has been postulated that toxic forms of Aβ are oligomeric (Shankar et al., 2008) and that Aβ may exert neurotoxic effect by forming amyloid plaques. The human APP Swedish mutation leads to increased β-secretase processing of APP and increased Aβ production (Citron et al., 1992, 1994; Johnston et al., 1994): these changes are recapitulated in *App^s/s^* knock-in rats, with Aβ40 and Aβ42, but not Aβ43, increasing equivalently in male and female *App^s/s^* rats (Figures 4, 5A) (Tambini et al., 2020). The Aβ42/Aβ40 ratio was comparable in *App^s/s^* rats and control *App^h/h^* animals of both sexes, and the Aβ43/Aβ40 ratio was reduced in both male and female *App^s/s^* rats compared to male and female *App^h/h^* animals (Figure 5A). Despite the increased production of Aβ42, we found no evidence supporting an increase in brain Aβ oligomers and the formation of amyloid pathology in *App^s/s^* rats of either sex (Figures 5B,C). In summary, Aβ levels are associated with the Behavioral Sequencing performances of female *App^s/s^* versus same-sex *App^h/h^* rats. Although it is possible that A11 may not detect all toxic oligomeric species, and/or that toxic Aβ is only present in brain areas governing the behavioral tasks analyzed and in the CA3/CA1 hippocampal subregion, the evidence that *App^h/h^* males perform like *App^s/s^* male and *App^s/s^* female rats, which express significantly higher levels of Aβ, but worse than *App^h/h^* control females, which express comparable levels Aβ. are not consistent with an Aβ-centric paradigm. Tau pathology and neuroinflammation are also linked to AD pathogenesis. Yet, we found no evidence of tau-related pathological changes and of neuroinflammation in our knock-in rats (Figures 6, 7). Overall, changes in Aβ metabolism, tau-pathology and neuroinflammation, *per se’*, do not associate with cognitive performances in a higher complexity task.

In the CNS, APP is mainly expressed in neurons (Guo et al., 2012) and, in synapses, it is mainly found in synaptic vesicles (SV) (Groemer et al., 2011; Del Prete et al., 2014). The *in vivo* brain interactome of APP reveals a network with SV proteins (Norstrom et al., 2010; Kohli et al., 2012), which is enabled by two APP domains, one cytosolic (JCasp) and one intra-vesicular (ISVAID) (Del Prete et al., 2014; Fanutza et al., 2015). Interfering with these interactions modulates glutamatergic SV release in an opposite fashion (Fanutza et al., 2015). Cleavage of APP by β-secretase in SV cuts the ISVAID region into two halves and abrogates the intra-vesicular interaction, promoting glutamate release via the cytosolic JCasp interacting domain. The evidence that β-secretase inhibitors causes strong reduction in the frequency glutamatergic SV release (Filser et al., 2014), that β-secretase KO mice show an increase in PPF ratio, which in the author’s words is indicative of a reduction in presynaptic release (Wang et al., 2008), and that *App^s/s^* KI rats, in which β-processing of APP is favored, show augmented glutamate release at SC–CA3 > CA1 pyramidal cell synapses (Tambini et al., 2019), supports this β-secretase and APP−dependent glutamate release (BAD−Glu) functional model. Based on these premises, we analyzed LTP, a long-lasting form of synaptic plasticity and a cellular model for learning and memory, in 12-month-old knock-in rats. Potentiation induced by the ⊖-burst stimulation (STP phase of LTP) was genotype-dependent because it was significantly reduced in both male and female *App^s/s^* rats compared to male and female *App^h/h^* controls (Figure 11A). In contrast, the late phase of LTP was determined by an interaction between genotype and sex since it was similarly reduced in *App^s/s^* rats of both sexes and in *App^h/h^* male rats compared to *App^h/h^* females (Figure 11C). Intriguingly, this genotype-dependent and sex-dependent pattern mirrors the one determining cognitive performance in the Behavioral Sequencing task (Figure 3C). Although the data shown here do not establish a cause-effect relationship between L-LTP and cognitive performances, it is plausible that L-LTP efficiency determines behavioral sequencing cognitive performance given the role of LTP in governing behavior, learning and memory. If this were the case, correcting the LTP deficit may have a disease-modifying effect.

Thus, L-LTP and higher order brain functions are both determined by matching interactions between sex-dependent and genotype-dependent (i.e., the Swedish mutation) factors. With regards to sex-dependent factor(s), it is possible that in females the pathogenic expression of the Swedish mutation is favored; alternatively, male-dependent factor(s) may confer resilience to the pathogenic mechanisms initiated by the Swedish mutation. With regards to genotype-dependent factors, the mechanism behind LTP alterations is not directly addressed in this study. However we can speculate about potential mechanisms by which the Swedish mutation contributes to the L-LTP and cognitive deficits. This mutation augments levels of β-processing APP metabolites -including sAPPβSw, βCTF and Aβ- and reduces α-processing APP metabolites -such as sAPPαSw, αCTF and P3 peptides. Any of these alterations may contribute to the observed deficits. If, for example, the increase in Aβ is the main pathogenic mechanism, targeting Aβ should be therapeutically efficacious. On the other hand, increased β-cleavage of Swedish APP in the ISVAID may directly prompt L-LTP and cognitive deficits via potentiation of the BAD-Glu pathway. If this were the case, reducing β-cleavage of APP at synapses and/or targeting facilitation of glutamate release via the JCasp domain of APP could prevent/corrects these deficits, while targeting Aβ clearance, oligomerization, spreading, deposition, and γ-secretase-dependent production would have no beneficial effects. It is worth mentioning that, as previously noted (Tamayev and D’Adamio, 2012; Tamayev et al., 2012), targeting β-cleavage of APP would be beneficial in both scenarios, albeit for different reasons. Another possibility to bear in mind is that the amino acid substitutions K670N/M671L of the Swedish haplotype may alter the function of several APP metabolites including full-length APP, sAPPα and sAPPβ. At any rate, more studies are needed to connect via cause-effect mechanisms these hypotheses to LTP alterations. Finally, although we previously reported that GABAergic mechanisms appear intact in young *App^s^* rats (Tambini et al., 2019), the possibility that alterations in GABA transmission affect LTP at this later age cannot be formally excluded since recordings were performed without GABA receptor antagonists.

Although sporadic AD is more prevalent in females (Beam et al., 2018), there are no reports indicating a female preference on disease progression of Swedish FAD patients, although the number of patients that carry the mutation is quite low. Hence, based on the sex-dependence of L-LTP and cognitive deficits described here, and on the preconceived idea that valid model organisms of AD must develop amyloid and/or tangle pathology, it could be argued that the data presented here are irrelevant to AD pathogenesis because our *App^s^* knock-in animals do not model AD. Few arguments may counteract this criticism: (1) the gene editing strategy used here allows for a genetic faithfulness to the human disease that other amyloid models (i.e., transgenic models) do not possess; (2) *App^s^* knock-in rats may develop amyloid and/or tangle pathology later in life; (3) the phenotype described here may be related to a prodromic phase of cognitive impairment, in which a sex preference may transpire. This sex preference may be no longer apparent at later stages of the disease and may have gone unnoticed in humans given the small numbers of Swedish FAD cases and the aggressivity of the disease’s progression.

In conclusion, this study shows that cognitive performance in a knock-in model of FAD is sex and *App*-genotype dependent. In addition, they point to a potential link between L-LTP values and cognitive decline. These data suggest that pathways leading to LTP dysfunction could be targeted for disease-modifying AD therapy.

## Data availability statement

The raw data supporting the conclusions of this article will be made available by the authors, without undue reservation.

## Ethics statement

This animal study was reviewed and approved by the Rutgers Institutional Animal Care and Use Committee (IACUC) (Protocol #201702513). All experiments were done according to policies on the care and use of laboratory animals of the Ethical Guidelines for Treatment of Laboratory Animals of the NIH. All efforts were made to minimize animal suffering and reduce the number of rats used.

## Author contributions

LD’A performed conceptual design of experiments and experiment’s integration, co-wrote the manuscript, and generated the animals tested. MY designed, performed, and analyzed electrophysiology recordings, and co-wrote the manuscript. TY prepared biochemical specimens for biochemical analysis and brains for IHC analysis, designed, performed, and analyzed immunoblots, dot blots, ELISAs, and co-wrote the manuscript. MT designed, performed, and analyzed Intellicage analyses and Abeta ELISA with TY, and co-wrote the manuscript. SZ and LB performed IHC experiments. All authors contributed to the article and approved the submitted version.
